# Vital Signs: Decrease in Incidence of Diabetes-Related End-Stage Renal Disease among American Indians/Alaska Natives — United States, 1996–2013

**DOI:** 10.15585/mmwr.mm6601e1

**Published:** 2017-01-13

**Authors:** Ann Bullock, Nilka Ríos Burrows, Andrew S. Narva, Karen Sheff, Israel Hora, Akaki Lekiachvili, Hannah Cain, David Espey

**Affiliations:** ^1^Division of Diabetes Treatment and Prevention, Indian Health Service, Rockville, Maryland; ^2^Division of Diabetes Translation, CDC; ^3^National Institute of Diabetes Digestive and Kidney Diseases, National Institutes of Health, Bethesda, Maryland; ^4^National Center for Chronic Disease Prevention and Health Promotion, CDC; ^5^Office for State, Tribal, Local & Territorial Support, CDC.

## Abstract

**Background:**

American Indians and Alaska Natives (AI/AN) have the highest diabetes prevalence among any racial/ethnic group in the United States. Among AI/AN, diabetes accounts for 69% of new cases of end-stage renal disease (ESRD), defined as kidney failure treated with dialysis or transplantation. During 1982–1996, diabetes-related ESRD (ESRD-D) in AI/AN increased substantially and disproportionately compared with other racial/ethnic groups.

**Methods:**

Data from the U.S. Renal Data System, the Indian Health Service (IHS), the National Health Interview Survey, and the U.S. Census were used to calculate ESRD-D incidence rates by race/ethnicity among U.S. adults aged ≥18 years during 1996–2013 and in the diabetic population during 2006–2013. Rates were age-adjusted based on the 2000 U.S. standard population. IHS clinical data from the Diabetes Cares and Outcomes Audit were analyzed for diabetes management measures in AI/AN.

**Results:**

Among AI/AN adults, age-adjusted ESRD-D rates per 100,000 population decreased 54%, from 57.3 in 1996 to 26.5 in 2013. Although rates for adults in other racial/ethnic groups also decreased during this period, AI/AN had the steepest decline. Among AI/AN with diabetes, ESRD-D incidence decreased during 2006–2013 and, by 2013, was the same as that for whites. Measures related to the assessment and treatment of ESRD-D risk factors also showed more improvement during this period in AI/AN than in the general population.

**Conclusion and implications for public health practice:**

Despite well-documented health and socioeconomic disparities among AI/AN, ESRD-D incidence rates among this population have decreased substantially since 1996. This decline followed implementation by the IHS of public health and population management approaches to diabetes accompanied by improvements in clinical care beginning in the mid-1980s. These approaches might be a useful model for diabetes management in other health care systems, especially those serving populations at high risk.

## Introduction

In the United States, diabetes is the leading cause of end-stage renal disease (ESRD), which is kidney failure treated with dialysis or transplantation (*1*). The prevalence of diabetes among American Indians/Alaska Natives (AI/AN) in the United States in 2012 (15.9%) was higher than that among non-Hispanic blacks (blacks) (13.2%), Hispanics (12.8%) or non-Hispanic whites (whites) (7.6%) during 2010–2012 (*2*). Diabetes accounts for 44% of new cases of ESRD (diabetes-associated ESRD [ESRD-D]) in the overall U.S. population and for 69% among AI/AN (*1*). Prevention or delay of ESRD-D involves control of blood pressure and blood glucose, early identification and monitoring of kidney disease, and use of angiotensin converting enzyme (ACE) inhibitors and angiotensin II receptor blockers (ARB) in patients with albuminuria (*3*,*4*). This report presents trends in ESRD-D incidence for AI/AN compared with other racial/ethnic groups, and discusses the probable factors that influenced the improvements observed in this population during 1996–2013.

## Methods

Medicare covers ESRD treatment for beneficiaries regardless of age and pays most of the cost of ESRD treatment in the United States (*1*). The U.S. Renal Data System (USRDS) is a surveillance system for ESRD based on clinical and claims data reports to the Centers for Medicare & Medicaid Services (CMS). Funded by the National Institute of Diabetes and Digestive and Kidney Diseases of the National Institutes of Health, the USRDS collects, analyzes, and distributes demographic and clinical data on patients being treated for ESRD, including the primary diagnosis or cause of kidney failure. Because most ESRD patients become eligible for Medicare coverage after 90 days of ESRD treatment, only data on patients who have been treated for at least 90 days are included in the data set (*1*).

For each year studied, USRDS data were used to determine the number of adults aged ≥18 years in the United States who began treatment (dialysis or kidney transplantation) for ESRD-D. Data were analyzed for AI/AN, white, black, and Asian racial groups, which include persons of Hispanic and non-Hispanic origin. Data for persons of Hispanic origin were analyzed separately.

ESRD-D incidence was calculated using the number of newly treated ESRD-D cases and two population estimates for each racial and ethnic group: 1) total population from the U.S. Census during 1996–2013, and 2) population with diagnosed diabetes during 2006–2013.

The number of AI/AN with diagnosed diabetes was calculated using age- and sex-specific prevalence estimates from the Indian Health Service (IHS) National Data Warehouse during 2006–2013 and multiplying them by annual bridged single race population estimates for AI/AN from the U.S. Census; 2006 was the first year for which consistent prevalence data are available. The IHS National Data Warehouse includes patient registration and encounter data from IHS facilities, tribally operated health programs, and urban Indian (I/T/U) health systems.* These facilities serve approximately 2.2 million AI/AN persons who belong to 567 federally recognized tribes in 36 states.^†^ Diabetes cases were identified using diagnosis codes 250.0–250.93 from the *International Classification of Diseases, Ninth revision, Clinical Modification*. Patients were considered to have diagnosed diabetes if they had at least two health care visits with a diabetes diagnosis code reported during the fiscal year (*5*). For the other racial and ethnic groups, estimates of the adult population with diagnosed diabetes (self-reported) were derived from the National Health Interview Survey.^§^

ESRD-D incidence rates were age-adjusted based on the 2000 U.S. standard population, and joinpoint regression was used to analyze trends (*6*,*7*). Each trend segment is described by an annual percentage change (APC) with a 95% confidence interval (CI), and the trend for the entire study period is described by the average annual percentage change (AAPC). The rate of change for linear trends was tested to determine whether it was significantly different from zero. Results were considered significant if the p value was <0.05.

Measures of care for AI/AN with diabetes were obtained from the IHS Diabetes Care and Outcomes Audit (Audit), including prescription of ACE inhibitors and ARBs; blood pressure; hemoglobin A1C to assess glucose control; and urine albumin-to-creatinine ratio testing for identifying and monitoring diabetic kidney disease. The Audit is an annual process for assessing diabetes care and health outcomes for AI/AN with diagnosed diabetes who receive care at I/T/U facilities, tracking performance on several dozen diabetes care measures and prevalence of several diabetes complications, including kidney disease.^¶^

## Results

**FIGURE 1:**
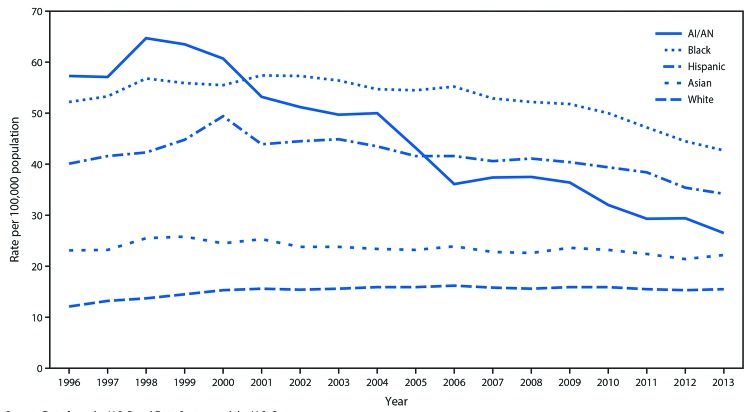
Incidence* of diabetes-related end-stage renal disease among adults aged ≥18 years, by race and ethnicity — United States, 1996–2013 **Source:** Data from the U.S. Renal Data System and the U.S. Census. **Abbreviation:** AI/AN=American Indians and Alaska Natives. * Rate per 100,000 population and age-adjusted based on the 2000 U.S. standard population. Racial groups include persons of Hispanic and non-Hispanic origin; Hispanics may be of any race.

Among AI/AN adults, age-adjusted ESRD-D incidence per 100,000 population increased, but not significantly, from 57.3 in 1996 to 63.5 in 1999 and then declined to 26.5 in 2013, a decrease of 54% (AAPC = −4.4% per year [95% CI = −5.7% to −3.0%], p<0.001) throughout the study period (Figure 1) (Table 1). Among other racial/ethnic groups, age-adjusted ESRD-D incidence among adults declined beginning in 1998 for Asians, 2001 for blacks, 2006 for whites, and 2000 for Hispanics.

**TABLE 1:** Age-adjusted incidence rates* and trend analysis of diabetes-related end-stage renal disease among adults aged ≥18 years in the general population (1996–2013) and in the diabetic population (2006–2013), by race and ethnicity^_†_^ — United States

General population	Rate		% change	Overall trend		Trend segment 1^§^			Trend segment 2/3^§^		
	1996	2013		AAPC (95% CI)	p value	Period	APC (95% CI)	p value	Period	APC (95% CI)	p value
AI/AN	57.3	26.5	−54	−4.4 (−5.7 to −3.0)	<0.001	1996–1999	3.3 (−4.7 to 12.0)	0.40	1999–2013	−6.0 (−6.7 to −5.2)	<0.001
Asians	23.1	22.2	−4	−0.2 (−1.0 to 0.6)	0.62	1996–1998	5.4 (−2.2 to 13.6)	0.15	1998–2013	−0.9 (−1.2 to −0.6)	<0.001
Blacks	52.2	42.7	−18	−1.3 (−1.8. to −0.7)	<0.001	1996–2001	1.7 (0.5 to 2.9)	0.01	2001–2009	−1.3 (−2.0 to −0.6)	0.002
									2009–2013	−4.8 (−6.4 to −3.2)	<0.001
Whites	12.1	15.5	+28	1.4 (0.9 to 1.8)	<0.001	1996–2000	5.8 (4.5 to 7.1)	<0.001	2000–2006	0.7 (−0.1 to 1.6)	0.09
									2006–2013	−0.6 (−1.1 to −0.1)	0.03
Hispanics	40.1	34.2	−15	−0.6 (−1.3 to 0.1)	0.08	1996–2000	4.4 (1.6 to 7.3)	0.005	2000–2013	−2.1 (−2.5 to −1.6)	<0.001

Diabetic population	Rate		% change	Overall trend		Trend segment 1^§^			Trend segment 2/3^§^		
	2006	2013		AAPC (95% CI)	p value	Period	APC (95% CI)	p value	Period	APC (95% CI)	p value
AI/AN	210.7	152.7	−28	−4.9 (−7.0 to −2.7)	<0.001	2006–2009	−2.0 (−8.2 to 4.7)	0.41	2009–2013	−7.0 (−10.8 to −3.0)	0.01
Asians	219.0	227.4	+4	−0.8 (−5.9 to 4.6)	0.72	2006–2013	−0.8^¶^ (−5.9 to 4.6)	0.72	—	—	—
Blacks	379.8	329.6	−13	−2.8 (−4.7 to −1.0)	0.01	2006–2013	−2.8^¶^ (−4.7 to -1.0)	0.01	—	—	—
Whites	185.8	159.0	−14	−2.0 (−3.9 to −0.0)	0.05	2006–2013	−2.0^¶^ (−3.9 to −0.0)	0.05	—	—	—
Hispanics	287.6	223.0	−22	−0.1 (−0.1 to −0.1)	<0.001	2006–2008	−0.3 (−0.5 to −0.1)	0.01	2008–2013	−0.0 (−0.0 to 0.0)	0.79

**Abbreviations:** AAPC = average annual percentage change; AI/AN = American Indians and Alaska Natives; APC = annual percentage change; CI = confidence interval.

*Per 100,000 population or per 100,000 diabetic population and age-adjusted based on the 2000 U.S. standard population.

^†^Racial groups include persons of Hispanic and non-Hispanic origin; Hispanics may be of any race.

^§^Trend segment identified by joinpoint regression.

^¶^APC = AAPC (i.e., trend had 0 joinpoints).

**FIGURE 2:**
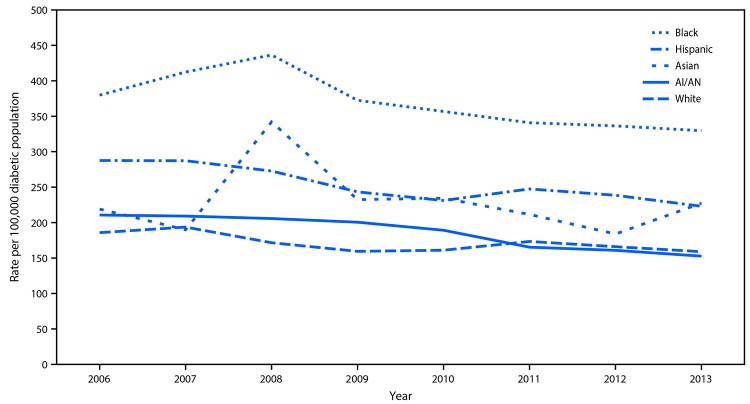
Incidence* of diabetes-related end-stage renal disease among adults aged ≥18 years with diabetes, by race and ethnicity — United States, 2006–2013 **Sources:** U.S. Renal Data System, U.S. Diabetes Surveillance System, and data from the Indian Health Service applied to the U.S. Census population. **Abbreviation:** AI/AN=American Indians and Alaska Natives. * Rate per 100,000 diabetic population and age-adjusted based on the 2000 U.S. standard population. Racial groups include persons of Hispanic and non-Hispanic origin; Hispanics may be of any race.

Among AI/AN adults with diabetes, ESRD-D incidence declined during 2009–2013 (APC = −7.0% per year [−10.8% to −3.0%], p = 0.01) and, by 2013, was similar to that of whites with diabetes (152.7 versus 159.0 per 100,000 diabetic population, p = 0.84) (Figure 2) (Table 1). Among other racial/ethnic groups with diabetes, ESRD-D incidence declined in blacks and in whites during 2006–2013, and showed no consistent trend among Asians. Among Hispanics, ESRD-D incidence declined during 2006–2008, and then leveled off.

**FIGURE 3:**
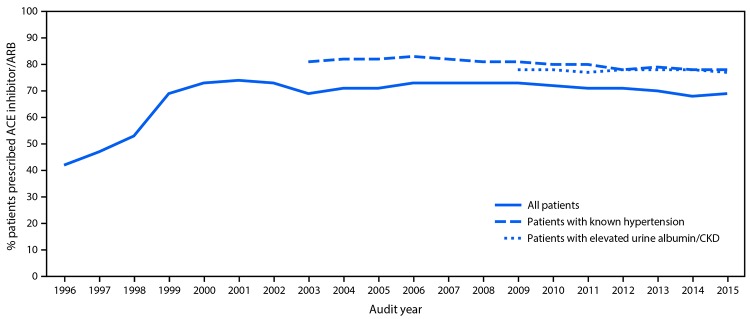
ACE Inhibitor/ARB prescription in AI/AN patients with diabetes, 1996–2015 **Source:** Indian Health Service Diabetes Care and Outcomes Audit. **Abbreviations:** ACE = angiotensin converting enzyme; AI/AN = American Indians and Alaska Natives; ARB = angiotensin receptor blocker; CKD = chronic kidney disease.

Data from the Audit show that prescription of ACE inhibitors and ARBs for AI/AN patients with diabetes increased substantially, from 42% in 1997 to 74% in 2002, and then remained steady, ranging from 68% to 73% each year through 2015 (Figure 3). Among AI/AN patients with diabetes and hypertension or chronic kidney disease (CKD), prescription of ACE inhibitors and ARBs was >77% for each year studied. Furthermore in 2014, among AI/AN with diabetes, 76% were prescribed ACE inhibitors or ARBs, compared with 56% of adults with diabetes in the general U.S. population during 2009–2014, assessed using National Health and Nutrition Examination Survey data (*8*).** Average blood pressure levels in AI/AN with diabetes have been well controlled since 1997, the first year such data were available. In 2015, average blood pressure among >101,000 AI/AN in the Audit with diabetes and hypertension was 133/76 mmHg, below the target of <140/90.^††^ Average hemoglobin A1C levels in AI/AN with diabetes decreased 10% from 1996 to 2014, from 9.0% to 8.1% (*9*). Finally, urine albumin-to-creatinine ratio testing was performed in 50% of AI/AN aged ≥65 years with diabetes in 2013, increasing to 62% by 2016. In the general Medicare diabetes population aged ≥65 years, the rate of urine albumin testing was 40% in 2013 (*1*).

## Conclusions and Comment

Among AI/AN adults, age-adjusted ESRD-D incidence decreased 54% during 1996–2013; by 2013, among adults with diabetes, the ESRD-D rate was the same in AI/AN as in whites. This decline is especially remarkable given the well-documented health and socioeconomic disparities in the AI/AN population, including poverty, limited health care resources, and disproportionate burden of many health problems (*10*). The findings in this report are consistent with other studies among AI/AN nationwide and among Pima Indians in the Southwest, which concluded that improvements in blood pressure, blood glucose, and the use of ACE inhibitors and ARBs played a significant role in the decline of ESRD-D in these populations (*11*,*12*).

The decrease of ESRD-D in AI/AN with diabetes was likely the result of improvements in both process and outcome measures presented in this report. Prescription of ACE inhibitors and ARBs in AI/AN with diabetes increased 76% from 1997 to 2002. In 2014, prescription of these medications among AI/AN with diabetes was 36% higher than for the overall U.S. population with diabetes (*8*). Similarly, among persons with diabetes aged ≥65 years, the rate of urine albumin-to-creatinine ratio testing is 55% higher in AI/AN compared with Medicare beneficiaries (*1*). Outcome measures are also positive, including blood pressure control in AI/AN with diabetes and hypertension and improved glycemic control overall. Establishing and sustaining these favorable trends in diabetes management and prevention of ESRD-D are related to population and team-based approaches to diabetes management undertaken by the IHS.

Starting in the mid-1980s, IHS implemented systematic approaches to diabetes care that have contributed to the outcomes presented here (*13*,*14*). These approaches were informed by public health and population management principles, which focus not just on short-term outcomes for individual patients who seek care, but also long-term outcomes, costs, disparities, and wellness of the entire community (*15*). These approaches include multidisciplinary team-based, coordinated clinical care and education, community outreach, and tracking of clinical process and outcomes data at the local, regional, and national levels (*9*).

This IHS system of diabetes care enabled I/T/U sites to successfully and consistently deliver evidence-based interventions that reduce ESRD-D risk factors. In 1986, IHS developed its first Diabetes Standards of Care to disseminate evidence-based recommendations aimed at improving diabetes care for AI/AN (*13*). These standards were revised in the early 1990s to include assessment and treatment of CKD (*16*). IHS was one of the first systems to establish routine reporting of the estimated glomerular filtration rate, yearly monitoring of urine albumin excretion, and prescription of ACE inhibitors and ARBs (*14*). Both of these classes of therapeutic agents have been shown to prevent or delay the development of ESRD-D in patients with albuminuria, independent of their effects in reducing blood pressure (*4*,*17*).

As data collection and analysis are fundamental components of an effective diabetes care system, IHS first implemented the Diabetes Care and Outcomes Audit in 1986 at several sites, and in 1997, developed a centralized, national database (*18*). Successful implementation of evidence-based clinical interventions as documented by the Audit might explain in part the decline in ESRD-D incidence in AI/AN adults with diabetes. IHS has made other improvements in diabetes care by developing clinical education programs and tools; culturally relevant patient education materials; and population-based management tools in the IHS electronic health record (*9*,*14*,*19*). I/T/U case managers help coordinate in-house care as well as referrals for specialty services, to facilitate greater care continuity than in more fragmented systems.^§§^ I/T/U facilities also support diabetes care and education by using public health nurses and community health workers to provide outreach and education to the community.^¶¶^^,^***

In 1997, Congress established the Special Diabetes Program for Indians (SDPI) (*9*). The SDPI provides much-needed funding to 301 I/T/U sites to implement interventions which reduce risk factors for diabetes and its complications, including ESRD-D (Table 2) (*9*).^†††^ In addition, SDPI funds have been used by IHS to improve its national program for disseminating evidence-based interventions and providing training, tools for data collection and analysis, and support to diabetes programs in AI/AN communities across the country. Because of SDPI, the partnership of IHS and I/T/U programs is stronger, and together they provide a comprehensive public health–oriented national program that has demonstrated success in addressing the diabetes epidemic and reducing complications such as ESRD-D (*9*).

**TABLE 2:** Percentage of Special Diabetes Program for Indians programs reporting diabetes services — United States

Intervention	1997 %	2013 %
Diabetes clinical teams	30	96
Diabetes patient registries	34	98
Nutrition services for adults	39	93
Access to registered dietitians	37	79
Access to physical activity specialists	8	74
Access to culturally tailored diabetes education materials	36	97

**Source:** Special Diabetes Program for Indians — 2014 report to Congress. Rockville, Maryland: Indian Health Service; 2014. https://www.ihs.gov/newsroom/includes/themes/newihstheme/display_objects/documents/RepCong_2016/SDPI_2014_Report_to_Congress.pdf.

The findings in this report are subject to at least five limitations. First, the data are for persons receiving ESRD treatment as reported to CMS and do not include patients who refused treatment, those who died before receiving treatment, or those whose treatment was not reported to CMS. Second, primary diagnosis was obtained from the CMS Medical Evidence Report and was based on a physician's assessment of the patient, which could be influenced by the physician’s awareness of diabetes prevalence among AI/AN. Third, differential classification of AI/AN race in the USRDS, U.S. Census, and IHS data systems could result in over- or underestimation of the actual incidence of ESRD-D in this population. Fourth, IHS data on diabetes prevalence might not be representative of the total AI/AN population and might result in over- or underestimation of the number of AI/AN with diabetes in the United States and, therefore, the incidence of ESRD-D. Although these biases might have affected incidence estimates, trends in incidence would not be affected if the biases remained consistent over time. Finally, the data on diabetes measures reflect care provided to AI/AN who access the I/T/U system and cannot be generalized to AI/AN who do not.

ESRD-D is a disabling and costly condition associated with high mortality.^§§§^ The Medicare expenditure per person per year for hemodialysis patients was $84,550 in 2013, and the per person per year cost for ESRD-D was $82,141 (*1*). In 2013, total Medicare spending for ESRD-D was $14 billion, about half (45%) of the $31 billion Medicare spending for ESRD overall (*1*). A decrease in ESRD-D incidence in the general U.S. population comparable to that experienced in the AI/AN population could result in fewer cases of newly treated ESRD-D and contribute to leveling or lowering of total Medicare expenditures for ESRD. Integrating public health, clinical, and community-based approaches to deliver evidence-based interventions aimed at reducing ESRD-D risk factors can sustain and improve trends in ESRD-D incidence.

**Key Points**

• In the United States, American Indians/Alaska Natives (AI/AN) are more likely to have diagnosed diabetes than any other racial or ethnic group. In response to the epidemic of diabetes in AI/AN, the Indian Health Service (IHS) developed a comprehensive diabetes program, which includes clinical care improvements as well as public health and population management approaches.

• End-stage renal disease (ESRD) is a costly complication of diabetes. Incidence of ESRD related to diabetes (ESRD-D) among AI/AN decreased 54% during 1996–2013. By 2013, in adults with diabetes, ESRD-D incidence was the same in AI/AN as in whites.

• Since diabetes and its complications are public health problems, the response of IHS, a direct care agency organized around a public health model, might be useful to other health care systems.

• Additional information is available at https://www.cdc.gov/vitalsigns.

## Footnotes

* https://www.ihs.gov/NDW.

† IHS. The 2016 Indian Health Service and Tribal Health Care Facilities’ Needs Assessment Report to Congress. https://www.ihs.gov/newsroom/includes/themes/newihstheme/display_objects/documents/RepCong_2016/IHSRTC_on_FacilitiesNeedsAssessmentReport.pdf.

§ CDC. U.S. Diabetes Surveillance System. http://www.cdc.gov/diabetes/data.

¶
https://www.ihs.gov/diabetes/audit/ .

** American Community Survey 1-Year Estimates, 2015. United States Census Bureau. https://www.census.gov/programs-surveys/acs/.

†† American Diabetes Association. Standards of Medical Care in Diabetes—2017. Diabetes Care 2017 Jan; 40 (Supplement 1): S1–S135.

§§ IHS: Special Diabetes Program for Indians—2011 report to Congress, 2011. https://www.ihs.gov/newsroom/includes/themes/newihstheme/display_objects/documents/RepCong_2012/2011RTC_Layout_10102012_508c.pdf.

¶¶ IHS. Public Health Nursing. http://www.ihs.gov/dper/index.cfm/planning/rrm/public-health-nursing.

*** IHS. Community Health Representatives. https://www.ihs.gov/chr.

††† IHS Division of Diabetes Treatment and Prevention. Special Diabetes Program for Indians FY 2016 Community-Directed Grant Programs 2016. https://www.ihs.gov/sdpi/includes/themes/newihstheme/display_objects/documents/factsheets/SDPI_FY2016_CD_GrantPrograms.pdf.

§§§ Morbidity and Mortality in Patients with Chronic Kidney Disease. USRDS Coordinating Center. https://www.usrds.org/2015/view/v1_03.aspx?zoom_highlight=mortality.
